# Predicting Changes of Body Weight, Body Fat, Energy Expenditure and Metabolic Fuel Selection in C57BL/6 Mice

**DOI:** 10.1371/journal.pone.0015961

**Published:** 2011-01-05

**Authors:** Juen Guo, Kevin D. Hall

**Affiliations:** Laboratory of Biological Modeling, National Institute of Diabetes and Digestive and Kidney Diseases, Bethesda, Maryland, United States of America; Fundació Institut Germans Trias i Pujol, Universitat Autònoma de Barcelona, CibeRES, Corporate Research Program on Tuberculosis, Spain

## Abstract

The mouse is an important model organism for investigating the molecular mechanisms of body weight regulation, but a quantitative understanding of mouse energy metabolism remains lacking. Therefore, we created a mathematical model of mouse energy metabolism to predict dynamic changes of body weight, body fat, energy expenditure, and metabolic fuel selection. Based on the principle of energy balance, we constructed ordinary differential equations representing the dynamics of body fat mass (FM) and fat-free mass (FFM) as a function of dietary intake and energy expenditure (EE). The EE model included the cost of tissue deposition, physical activity, diet-induced thermogenesis, and the influence of FM and FFM on metabolic rate. The model was calibrated using previously published data and validated by comparing its predictions to measurements in five groups of male C57/BL6 mice (N = 30) provided *ad libitum* access to either chow or high fat diets for varying time periods. The mathematical model accurately predicted the observed body weight and FM changes. Physical activity was predicted to decrease immediately upon switching from the chow to the high fat diet and the model coefficients relating EE to FM and FFM agreed with previous independent estimates. Metabolic fuel selection was predicted to depend on a complex interplay between diet composition, the degree of energy imbalance, and body composition. This is the first validated mathematical model of mouse energy metabolism and it provides a quantitative framework for investigating energy balance relationships in mouse models of obesity and diabetes.

## Introduction

The mouse has become the most popular model organism for investigating the molecular mechanisms regulating energy metabolism and body weight (BW). However, a quantitative understanding of energy expenditure in mice remains lacking as highlighted by recent articles addressing problems with the interpretation of indirect calorimetry measurements [1]–[4]. Indeed, it is often unclear whether an observed BW change in mice is a result of altered energy intake (EI), energy expenditure (EE), or both. While we know that diet and EE impact metabolic fuel selection and body fat change over time, their quantitative relationship is uncertain. From a physiological perspective, a proper understanding of the metabolic phenotypes of various mouse models requires quantitative integration of these variables and how they change over time.

To begin addressing these issues, we present a mathematical model of EE and metabolic fuel selection in male C57BL/6 mice. Our EE model incorporated the influence of body fat mass (FM), fat-free mass (FFM), the energy cost of tissue deposition, physical activity, and diet-induced thermogenesis (DIT). We combined the EE model with a mathematical model of energy partitioning to predict changes of BW, FM, and respiratory quotient (RQ) in response to measured changes of food intake. The model was validated by accurately predicting the BW and FM data from an independent set of experiments in C57BL/6 mice without adjusting any model parameters. The mathematical model demonstrates the complex relationships between metabolic fuel selection, diet composition, energy imbalance, and body composition change and provides a quantitative framework for investigation of murine energy metabolism.

## Methods

### Modeling Energy Expenditure and Body Composition Change

We begin with the law of energy conservation, also known as the energy balance equation:
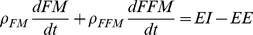
(1)where 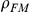 = 9.4 kcal/g and 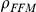 = 1.8 kcal/g are the energy densities for changes in FM and FFM, respectively [5]. EI is the total metabolizable energy intake rate corrected for spillage. We assumed that the calculated metabolizable energy intake based on food intake measurements adequately accounted for any differences of digestibility between the diets. We did not directly measure the energy content of the feces to confirm this assumption.

We previously showed that there is a well-defined, time-invariant function, *α*, that describes the relationship between changes of FFM and FM in adult male C57BL/6 mice:
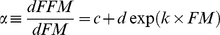
(2)where the parameters *c* = 0.1, *d* = 1.89×10^−4^, and *k* = 0.45 g^−1^ specify the shape of the empirically measured function *α* [6]. This function allows us to write equation 1 as a pair of differential equations specifying the rates of change of FM and FFM [6], [7]:
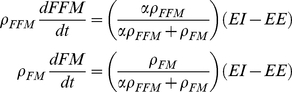
(3)


Given measurements of EI, solving equation 3 requires a model of EE which was adapted from published human models [8]–[10]:
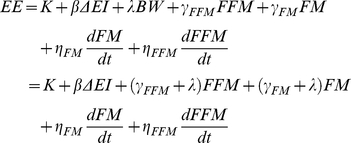
(4)where *K* is a thermogenesis parameter which was assumed to be constant for a fixed environmental temperature and λ represents physical activity whose energy cost was assumed to be proportional to BW. The values for K and λ were determined in the model calibration procedure described below. The parameter *β* accounts for the thermic effect of feeding as well as adaptive changes of EE as a result of diet changes. We will refer to the product *β*ΔEI as diet-induced thermogenesis (DIT) where ΔEI is the change of energy intake compared to baseline chow and the value *β*  = 0.4 was determined in a recent analysis of rodent feeding and body composition data [11]. The parameters η_FM_  = 0.18 kcal/g and η_FFM_  = 0.23 kcal/g account for the biochemical efficiencies associated with fat and protein synthesis [11] assuming that the change of fat-free mass is primarily accounted for by body protein and its associated intracellular water [5].

The parameters *γ_FFM_* and *γ_FM_* determine how metabolic rate varies with FFM and FM, respectively. To estimate the values of *γ_FFM_* and *γ_FM_*, we note that basal metabolic rate (BMR) across species is well described by the Kleiber 3/4 power law of BW:

*BMR ∼ BW* ^¾^ [12]. Within a species, FFM and FM are proportional to BW. Therefore,
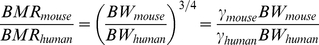
(5)and
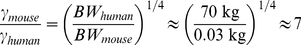
(6)


Using equation 6 to rescale the human values of *γ_FFM_*  = 22 kcal/kg/d and *γ_FM_*  = 3.6 kcal/kg/d [13] results in mouse values of *γ_FFM_*  = 0.15 kcal/g/d and *γ_FM_*  = 0.03 kcal/g/d.

### Data for Model Calibration

**Figure 1:**
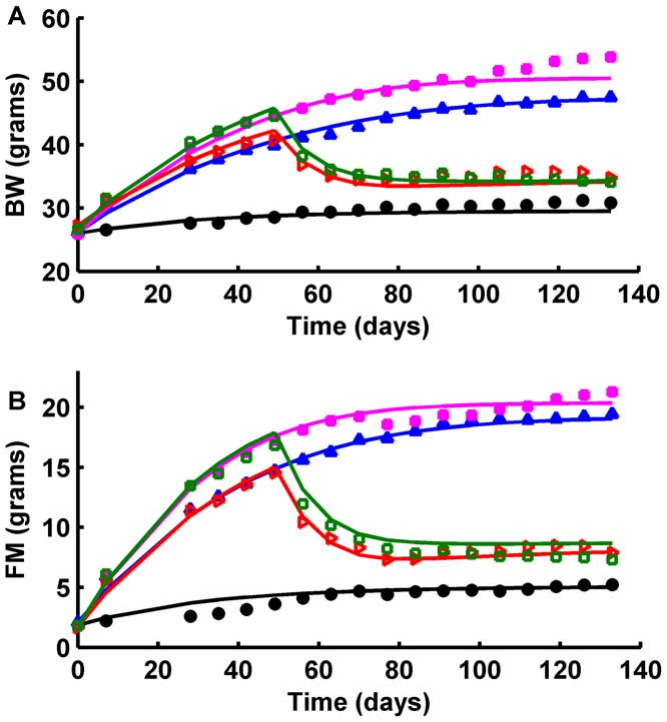
Mathematical model calibration. BW (A) and FM (B) data were obtained from 47 male C57BL/6 mice that were randomly assigned to five groups: the C group (solid circles) on a chow diet; the HF group (solid blue triangles) on a high fat diet; the EN group (solid magenta squares) on a high fat diet plus liquid Ensure; the HF-C group (open red triangles) switched from high fat to chow after 7 weeks; and the EN-C group (open green squares) switched from high fat plus Ensure to chow after 7 weeks. The solid curves are the mathematical model fit to these data. Error bars have been suppressed for clarity.

The calibration data were obtained from a previously described study, the results of which are depicted in Figure 1 [14]. Briefly, we studied 47 three-month-old male C57BL/6 mice that were individually housed at a temperature of 22°C and randomly assigned to five groups: 1) C group (N = 12) on a chow diet (24% protein, 12% fat, and 64% carb.); 2) HF group (N = 12) on a high fat diet (14% protein, 59% fat, 27% carb.); 3) EN group (N = 11) on a high fat diet plus liquid Ensure® (14% protein, 22% fat, 64% carb.); 4) HF-C group (N = 6) switched from high fat to chow after 7 weeks; 5) EN-C group (N = 6) switched from high fat plus Ensure® to chow after 7 weeks. All animals received free access to water and food throughout the study. The high fat diet was provided using Rodent CAFÉ feeders (OYC International, Inc., MA), and liquid Ensure was provided in a 30-ml bottle with a rodent sip tube (Unifab Co., MI) and liquid intake was measured every day. Solid food intake was corrected for any visible spillage and was measured every day for the high fat diet and every other day for the chow diet using a balance with a precision of 0.01 g (Ohaus model SP402). Body composition was measured using 1H NMR spectroscopy (EchoMRI 3-in-1, Echo Medical Systems LTD, Houston, TX) and was recorded longitudinally throughout the study along with food intake and BW. The BW and FM at the beginning of the study were used as the initial values for the model inputs.

We certify that all applicable institutional and governmental regulations concerning the ethical use of animals were followed during this research. All procedures were approved by the National Institute of Diabetes and Digestive and Kidney Diseases Animal Care and Use Committee under protocol K009-LBM-07.

### Model Calibration Procedure

We assumed that the physical activity parameter λ depended on the diet as described by the following differential equation:
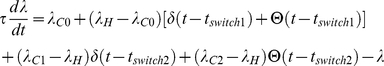
(7)where the initial value λ(0)  =  λ*_C0_* was the physical activity for the baseline chow diet group, C. For the HF, EN, HF-C and EN-C mice that were switched to a high energy diet at time *t_switch1_*, the physical activity parameter was assumed to change immediately to the value λ*_H_* (as implemented using the Dirac delta function, *δ*) and to remain at this level during the high energy diet (as implemented using the Heaviside function, Θ). For the HF-C and EN-C mice switched back to the chow diet at time *t_switch2_* we assumed that they immediately changed their physical activity to the value λ*_C1_* which then exponentially relaxed to a value of λ*_C2_* with a time constant of τ  = 14 days.

The model equations were numerically solved with the ODE45 solver using MATLAB software version R2008a (http://www.mathworks.com). While most model parameters were defined from analysis of previous data as stated above and tabulated in Table 1, the parameters *K*, λ*_C0_*, λ*_C1_*, λ*_C2_*, and λ*_H_* had to be estimated using data from our calibration experiment. The parameter values were determined using the Markov Chain Monte Carlo method where random guesses for the parameter values are proposed along with a simple rule for updating the parameter values depending on how closely the model results match the data [15]. Specifically, the model was run for 100,000 rounds for each group of mice and the first 30000 were discarded as a burn-in period with one fifth of the subsequent rounds were retained. Parameter sets were drawn from a proposal density that was normally distributed and centered on the previous value. The variance of the proposal density was tuned for an average acceptance rate of around 0.25 during the burn-in period. The convergence of the chain was assessed both by visual inspection of the trace plots for all the parameters and through the Geweke test [16]. At each sampling, the probability of accepting the new parameter set given current parameter set was min(1, r), where r is the Metropolis ratio. The energy intake in each group of animals was normally distributed with a standard error of 0.39, 0.39, 0.41, 0.55, and 0.55 Kcal/d for the C, HF, EN, HF-C, and EN-C groups, respectively. The 95% confidence intervals of the predicted energy output were obtained by calculating the 2.5th and 97.5th percentiles of the posterior distribution of energy output.

**Table 1:** Mathematical model symbols and parameter values.

Symbol	Description	Role	Value	Units
*I_F_*	Metabolizable fat intake	Input		kcal/d
*I_P_*	Metabolizable protein intake	Input		kcal/d
*I_C_*	Metabolizable carbohydrate intake	Input		kcal/d
*FM*	Body fat mass	Output		g
*FFM*	Fat-free mass	Output		g
*EE*	Total energy expenditure rate	Output		kcal/d
*RQ*	Respiratory quotient	Output		—
*ρ_FM_*	Energy density of fat mass changes	Parameter	9.4	kcal/g
*ρ_FFM_*	Energy density of lean mass changes	Parameter	1.8	kcal/g
*γ_FM_*	Metabolic rate of body fat mass	Parameter	0.03	kcal/g/d
*γ_FFM_*	Metabolic rate of fat-free mass	Parameter	0.15	kcal/g/d
*η_FM_*	Deposition cost for body fat	Parameter	0.18	kcal/g
*η_FFM_*	Deposition cost for fat-free mass	Parameter	0.23	kcal/g
*β*	Diet-induced thermogenesis	Parameter	0.4	—
λ*_C0_*	Physical activity at baseline	Parameter*	0.22	kcal/g/d
λ*_C1_*	Physical activity immediately after switching from high fat to chow	Parameter*	0.27	kcal/g/d
λ*_C2_*	Physical activity at steady state after switching from high fat to chow	Parameter*	0.19	kcal/g/d
λ*_H_*	Physical activity on a high fat diet	Parameter*	0.13	kcal/g/d
*K*	Basal thermogenesis rate	Parameter*	2.1	kcal/d

The parameters indicated with ‘*’ were determined by the model calibration procedure to minimize the difference between the simulated and observed BW and FM changes in the calibration experiment. No parameter values were adjusted for the validation experiment.

### Model validation

**Figure 2:**
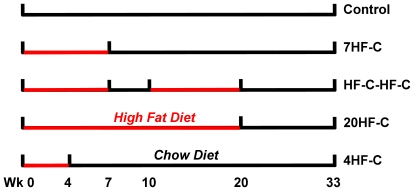
Experimental groups used for model validation. Mice were assigned to five different groups and provided *ad libitum* access to either a standard chow diet (black) or a high fat diet (red) for varying durations: 1) the control group on a chow diet (N = 6); 2) the 7HF-C group on a high fat diet for 7 wk followed by a switch to chow (N = 6); 3) the HF-C-HF-C on a high fat diet for 7 wk followed by a switch to chow for 3 wk, back on the high fat diet for 10 wk and followed by a switch back to chow (N = 6); 4) the 20HF-C on a high fat diet for 20 wk followed by a switch to chow (N = 6); 5) the 4HF-C group on high fat diet for 4 wk followed by a switch to chow (N = 6).

The model was validated using data from an independent study where 30 three-month-old male C57BL/6 mice were individually housed at a temperature of 22°C and randomly assigned to five *ad libitum* fed groups depicted in Figure 2: 1) the control group on a chow diet (N = 6); 2) the 7HF-C group on a high fat diet for 7 wk followed by a switch to chow (N = 6); 3) the HF-C-HF-C on a high fat diet for 7 wk followed by a switch to chow for 3 wk, back on the high fat diet for 10 wk and followed by a switch back to chow (N = 6); 4) the 20HF-C on a high fat diet for 20 wk followed by a switch to chow (N = 6); 5) the 4HF-C group on high fat diet for 4 wk followed by a switch to chow (N = 6). The chow and high fat diets were identical to the diets used in the calibration study. The body composition (Tables S1 and S2) and energy intake data (Table S3) are provided as Supporting Information.

The measured food intake rates were used as model inputs to predict BW and FM in the validation experiment and no model parameter values were adjusted. The energy intake in each group of animals was normally distributed with a standard error of 0.42, 0.48, 0.50, 0.53, and 0.55 kcal/d for the five groups, respectively. The 95% confidence intervals of the predicted energy output were obtained by calculating the 2.5th and 97.5th percentiles of the posterior distribution of EE.

### Metabolic Fuel Selection and the Respiratory Quotient

Since we are also interested in metabolic fuel selection, we considered the fates of dietary macronutrients including their oxidation rates, storage in the body, as well as major inter-conversion fluxes de novo lipogenesis (DNL) and gluconeogenesis (GNG). The following macronutrient balance equations represented these changes:
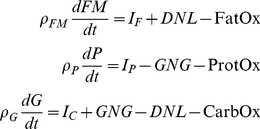
(8)where P is body protein, G is glycogen, and I_F_, I_P_ and I_C_ are the metabolizable intake rates of dietary fat, protein and carbohydrate, respectively. The oxidation rates of fat, protein, and carbohydrate (FatOx, ProtOx, and CarbOx, respectively) sum to EE.

To simplify the macronutrient balance equations, we note that glycogen stores are small, especially when compared with daily carbohydrate intake rates [6]. Thus, over the time-scale of interest the system is in a state of average carbohydrate balance:
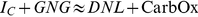
(9)


Therefore,
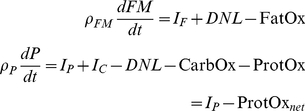
(10)where the net oxidation rates were defined as:
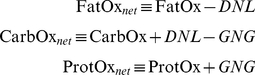
(11)


Assuming that FFM is proportional to body protein:
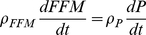
(12)


then equations 3, 8 and 9 resulted in the following expressions for the net macronutrient oxidation rates based on the model variables:
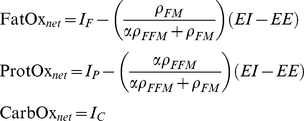
(13)


The respiratory quotient, RQ, is the carbon dioxide production rate divided by the oxygen consumption rate and was approximated by:
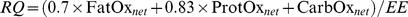
(14)


The calculated RQ may have slight inaccuracies during rapid transitions immediately after diet switches but will be reasonably accurate thereafter since the net carbohydrate oxidation rate is approximately equal to the carbohydrate intake rate on long time scales (several days in mice). Using the measured food intake rates along with the model calculated values of EE and FM, we applied equations 13 and 14 to calculate the dynamic changes of metabolic fuel selection over time. Note that these calculations did not influence the main model predictions for BW and FM.

We compared the calculated RQ values with the food quotient, FQ, which is a measure of the expected carbon dioxide production rate divided by the oxygen consumption rate had the food itself been combusted directly:
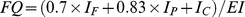
(15)


In a state of energy and macronutrient balance, RQ  =  FQ and any deviations from this equality reflect a situation where the metabolic fuel mixture differs from the diet and body composition changes will therefore result.

## Results

### Model Calibration

The comparison between the model simulations and the measured values for BW and FM from the calibration study is presented in Figures 1A and 1B, respectively. As previously described [14], the C group that only ate chow slightly increased BW and FM (solid circles in Figures 1A and 1B, respectively) throughout the study. The animals on the high fat diet (HF group shown in solid blue triangles) gained a substantial amount of weight and fat and the animals supplemented with the liquid Ensure (EN group shown in solid magenta squares) gained even more. Both the HF-C and EN-C groups (open red triangles and green squares, respectively) rapidly lost weight and fat after the switch from the high energy diets to the chow diet and reached a stable BW and FM four weeks after the diet switch at a level significantly higher than the C group.

The model simulations, depicted by the solid curves in Figure 1, agreed with the measurements for both the BW and FM measurements thereby demonstrating that the model was able to accurately describe the observations in the calibration study. The best-fit model parameters for physical activity were λ*_H_*  = 0.13 kcal/g/d for the HF and HF-C groups and λ*_H_*  = 0.16 kcal/g/d for the EN and EN-C groups (note that the latter value for λ*_H_* was irrelevant for the validation study since Ensure was not used). The baseline physical activity was λ*_C0_*  = 0.22 kcal/g/d for all groups, and following the switch to chow in the HF-C and EN-C groups λ*_C1_*  = 0.27 kcal/g/d and λ*_C2_*  = 0.19 kcal/g/d. Thus, introduction of the high fat diet resulted in a predicted drop of physical activity by ∼40% below the baseline chow diet. When the diet was switched back to chow, there was an immediate increase of physical activity, reaching ∼20% higher than baseline, which subsequently relaxed back to a value close to baseline. Since all mice were individually housed and kept at the same 22°C environmental temperature, the thermogenesis parameter *K*  = 2.1 kcal/d was constant across all groups. The EE and RQ predictions closely matched the previously published estimates of these variables (not shown) [6].

### Model Validation

**Figure 3:**
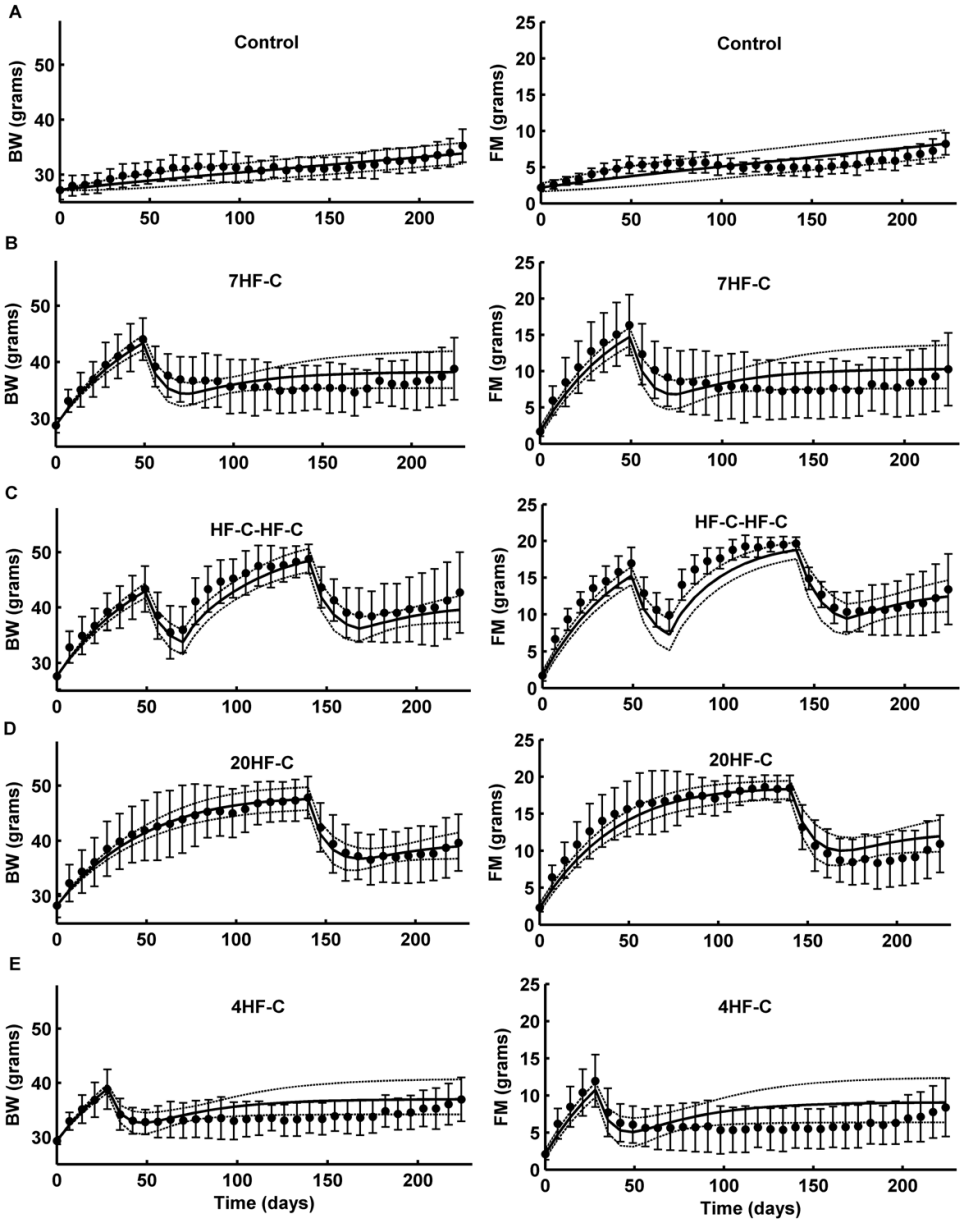
Mathematical model validation. Model simulations (solid black curves; dashed curves are 95% confidence intervals) were compared with data on BW (left column) and FM (right column) obtained from 30 male C57BL/6 mice randomly assigned to five groups: A) the control group on a chow diet; B) the 7HF-C group on a high fat diet for 7 wk followed by a switch to chow; C) the HF-C-HF-C on a high fat diet for 7 wk followed by a switch to chow for 3 wk, back on the high fat diet for 10 wk, followed by a switch back to chow; D) the 20HF-C on a high fat diet for 20 wk followed by a switch to chow; and E) the 4HF-C group on high fat diet for 4 wk followed by a switch to chow. No model parameters were adjusted to fit these data.

Using the same parameter values determined in the calibration procedure, Figure 3 shows that the model (curves) accurately predicted the measurements (•) of BW and FM of the five validation groups shown in the left and right columns, respectively. The 95% confidence intervals predicted by the model (dashed curves) and the error bars of the measurements overlapped throughout the entire study. Moreover, most of the model predictions fell within one standard error of the observed data for both BW and FM.

**Figure 4:**
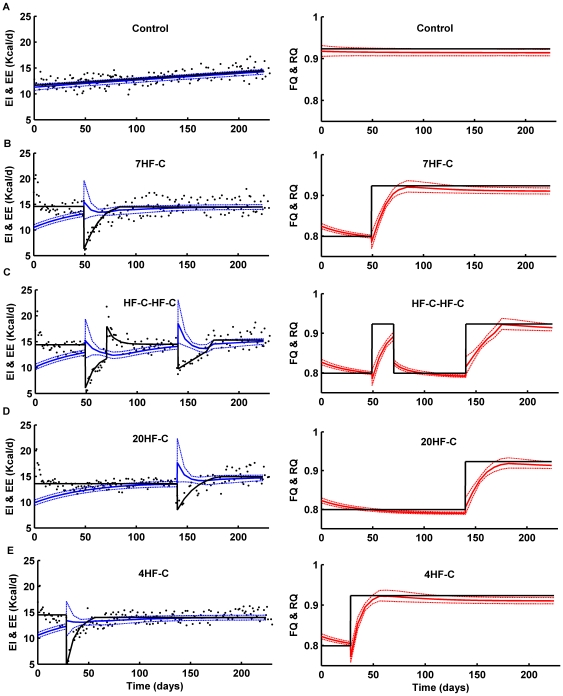
Mathematical model predictions of EE (left column) and metabolic fuel selection (right column). In the left column, the data points (•) are the EI measurements for the experiment depicted in Fig. 2 which were fit to the solid black curves and used as model inputs. The solid blue curves are the model predicted EE with the dashed blue curves representing the 95% confidence intervals. In the right column, the solid black curves represent the FQ of the diets and the solid red curves are the model predicted RQ with the dashed red curves representing the 95% confidence intervals.

The dynamics of EI and EE for the various groups are presented in the left column of Figure 4. The measured EI data (•) were first fit by the solid black curves which were used as the model inputs. The simulated EE dynamics are depicted using solid blue curves with the 95% confidence intervals shown using dashed blue curves. The control group EE was only slightly lower than EI, which is consistent with the slow increase of BW and FM in this group (Fig. 4A). High fat feeding decreased EE at the beginning of the experiment compared with the control group which, along with increased EI, resulted in significant weight gain that was accompanied by a gradual increase of EE. In the 7HF-C and HF-C-HF-C groups (Fig. 4B and 4C), switching from the high fat diet to chow at week 7 caused a small transient increase of EE due to increased physical activity which, along with the dramatic fall of EI, gave rise to the rapid BW losses shown in Fig. 3B and 3C. In contrast to the small increase of EE observed at week 7 upon switching to chow in the 7HF-C and HF-C-HF-C groups, the model predicted a substantially greater increase of EE after the diet switches at week 20 in the HF-C-HF-C and 20HF-C groups (Fig. 4C and 4D). This was predicted to result from the same transient increase of the physical activity parameter, λ, leading to a greater increase of EE since the BW was higher at week 20 versus week 7 and the energy cost of physical activity is given by the product λ×BW. Furthermore, the drop of EI was not as great upon switching to chow at week 20 versus week 7 and therefore the decrease of DIT did not offset the increased physical activity to the same extent as it did at 7 weeks. In the HF-C-HF-C group, although an increase in EI was observed at week 10 when the animals were switched from chow diet back to high fat diet, the EE remained relatively unchanged which resulted in rapid weight regain. The relative stability of EE at week 10 was predicted to result from a balance between the decreased physical activity on the high fat diet which was completely offset by the increased DIT.

The predicted dynamics of RQ (red) and FQ (black) are presented in the right column of Figure 4. The control group had RQ approximately equal to FQ  = 0.92 corresponding to the chow diet. The high fat diet had a lower FQ  = 0.80 and RQ immediately dropped at the onset of high fat feeding to a value slightly higher than FQ. Subsequently, RQ gradually fell as FM increased. When the animals were switched from the high fat diet to chow at week 7, a transient decrease of RQ was predicted right after the diet switch in the 7HF-C and HF-C-HF-C groups (Fig. 4B and 4C) and a similar transient decrease of RQ was seen in the 4HF-C group at week 4 (Fig. 4D). However, such a decrease of RQ was not predicted when switching from high fat to chow at week 20 in the 20HF-C and HF-C-HF-C groups because their higher FM had already dropped the RQ to a lower value at the time of the diet switch and the decrease of EI was not as great at week 20. When the HF-C-HF-C group was switched from the chow diet to the high fat diet at week 10, an immediate decrease of RQ was predicted similar to the initial onset of high fat feeding at week 0. These results highlight the complex interplay between diet composition, the degree of energy imbalance, and the body composition in determining metabolic fuel selection.

## Discussion

To our knowledge, there is only one previous report of a mathematical model of mouse metabolism and BW regulation and that model focused on the role of leptin to influence both EI and EE [17]. While that previous model led to interesting theoretical insights regarding feedback control of BW, the mathematical model was not validated and did not address the issue of metabolic fuel selection [18]. Our previous mathematical analysis used measured BW and food intake data as model inputs to estimate EE and fuel selection dynamics [6]. Here, we extended our previous analysis by explicitly modeling the determinants of EE. The only model input was food intake and this information allowed our model to accurately predict the observed changes of BW and FM in five independent groups of animals. Therefore, our model is the first validated mathematical model of mouse energy metabolism.

The model also predicted dynamic changes of RQ and EE over the entire course of the study based on the principle of energy conservation. However, we were unable to directly confirm these predictions since we did not have corresponding indirect calorimetry data. Such measurements would be prohibitive over the entire time course of the study, and measurements at isolated time points would likely lead to behavior change in the animals as they are removed from their home cages. Indeed, a recent elegant study demonstrated that the indirect calorimetry procedure caused weight loss mice that had previously been gaining weight on either a high fat or chow diet [19]. Weight gain only occurred when mice previously fed chow were provided with a novel high fat diet in the indirect calorimetry chamber. While the authors presented a simple statistical method to adjust the indirect calorimetry measurements based on the observed weight changes [19], the fact remains that the behavior of the animals during the procedure was clearly not representative of the extended study duration. Our previously described methodology for estimating RQ and EE avoids this difficulty [6], and the comparison of our model predictions for RQ and EE closely matched our previous estimates (not shown).

Regardless of the likely behavior changes introduced by the procedure, indirect calorimetry may be useful for investigating the contribution of FFM and FM to EE and we compared our mathematical model of EE with the results from a recently published mouse study [4]. Our equation 4 predicts that the coefficients describing how total EE varies with FFM and FM have values of (*γ_FFM_* + λ)  = 0.23 cal/g/min and (*γ_FM_* +λ)  = 0.14 cal/g/min averaged across all of the diets used in the present study. These values agree reasonably well with the regression coefficients of 0.269 cal/g/min and 0.144 cal/g/min found by Kaiyala et al. using mice fed various diets where total EE was regressed against FFM and FM, respectively [4]. Thus, these data lend further independent support to our model which also includes the influence of DIT, tissue deposition costs, and physical activity in addition to the dependence on FM and FFM.

We assumed that physical activity depended on the diet composition which is in accordance with previous studies showing that high fat diets result in substantial decreases of physical activity [14], [20]. Interestingly, to match the BW data from our calibration study our model predicted that high fat feeding caused an immediate and sustained decrease of physical activity whereas switching back to a chow diet causes a transient overshoot of physical activity, which together with a dramatic reduction of EI, resulted in rapid weight loss. This is consistent with previous studies showing that caloric restriction leads to increased activity in mice [21], but in our study the mice voluntarily restricted their own intake of chow after the high fat diet was removed. The degree of increase of total EE upon switching to the chow diet was found to depend on BW at the time of the diet switch. Furthermore, the decrease of EI caused a decrease of DIT that offset the effect of increased physical activity.

Our model has several limitations. First, the time scale of the model is days, weeks and months and therefore does not address within-day dynamics such as the transition from fed to fasted states. Second, physical activity behavioral changes need to be either input directly or calibrated from previous data for mice in similar environments. Therefore, applying the model calibrated for individually housed mice without running wheels will not necessarily represent the behavior of mice housed under different conditions (e.g., with running wheels). Similarly, all of our studies were conducted using individually housed mice kept at the same 22°C environmental temperature, but it is well-known that temperature can significantly impact EE in mice [22], [23]. This effect could be incorporated in our model by replacing the constant thermogenesis parameter *K* with a decreasing function of temperature until thermo neutrality is reached. It may also be necessary to make other model parameters temperature dependent. For example, the DIT parameter β and the dependence of metabolic rate on body fat, *γ_FM_*, since it is possible that UCP-1 activation in brown adipose tissue is temperature dependent and mediates DIT [24], although this effect is controversial [25].

We anticipate that modification of the model parameters will be required to appropriately represent other strains of mice, genetic knockouts, or transgenic mouse models. Indeed, the process of determining the parameter modifications required to accurately simulate different mouse models will provide important quantitative information regarding their integrative metabolic phenotypes and the differences between mouse models.

Our mathematical model provides a quantitative framework for integrating murine data on food intake, body weight, and body fat to help understand the complex dynamic relationships between diet, expenditure, body composition and metabolic fuel selection. In the future, we hope to validate our RQ and EE model predictions by comparing with indirect calorimetry data where mice spend many weeks inside suitably modified metabolic cages. We also plan to incorporate a more mechanistic representation of metabolic flux regulation and the influence on EE and metabolic fuel selection as was recently described in more detailed mathematical models of human metabolism published by our research group [26], [27]. Finally, we plan to include the potential influence of circulating factors such as insulin and leptin and eventually “close the loop” by modeling the regulation of food intake in mice.

## Supporting Information

Table S1 Body weight measurements and 95% confidence intervals.
(DOC)

Table S2 Body fat measurements and 95% confidence intervals.
(DOC)

Table S3 Energy intake measurements.
(DOC)
