# Supplementary material for: Predicting Changes of Body Weight, Body Fat, Energy Expenditure and Metabolic Fuel Selection in C57BL/6 Mice
Source: PLoS One. 2011 Jan 5;6(1):e15961. doi: 10.1371/journal.pone.0015961 (PMC3016341; doi:10.1371/journal.pone.0015961)
Supplement: Table S1 — Body weight measurements and 95% confidence intervals. (DOC) [file pone.0015961.s001.doc]

| day | BW, g |  |  |  |  | 95% CI of BW, g | | |  |  |
| --- | --- | --- | --- | --- | --- | --- | --- | --- | --- | --- |
|  | Control | 7HF-C | HF-C-HF-C | 20HF-C | 4HF-C | Control | 7HF-C | HF-C-HF-C | 20HF-C | 4HF-C |
| 0 | 27.2 | 28.7 | 27.6 | 28.2 | 29.3 | 1.7 | 1.3 | 2.4 | 2.1 | 0.7 |
| 7 | 28.0 | 33.1 | 32.9 | 32.3 | 32.9 | 1.8 | 2.0 | 2.9 | 3.3 | 1.6 |
| 14 | 28.2 | 35.1 | 34.9 | 34.4 | 35.2 | 1.7 | 3.0 | 3.4 | 3.9 | 2.6 |
| 21 | 28.5 | 36.9 | 36.7 | 36.1 | 36.9 | 1.8 | 3.1 | 3.1 | 4.4 | 3.2 |
| 28 | 29.2 | 39.5 | 39.2 | 38.5 | 38.8 | 1.8 | 4.0 | 3.3 | 5.0 | 3.7 |
| 35 | 29.8 | 41.0 | 40.0 | 39.9 | 34.2 | 2.1 | 3.9 | 3.8 | 5.0 | 2.9 |
| 42 | 30.0 | 42.6 | 41.8 | 41.1 | 33.0 | 2.0 | 4.2 | 4.0 | 5.1 | 2.4 |
| 49 | 30.3 | 44.1 | 43.3 | 41.9 | 32.8 | 2.0 | 3.7 | 4.2 | 5.5 | 2.3 |
| 56 | 30.7 | 39.2 | 38.5 | 42.6 | 32.8 | 2.0 | 3.6 | 4.2 | 6.2 | 2.9 |
| 63 | 31.3 | 37.6 | 35.5 | 43.0 | 33.2 | 2.3 | 3.8 | 4.8 | 6.0 | 3.3 |
| 70 | 31.1 | 36.9 | 36.0 | 43.9 | 33.1 | 2.1 | 4.0 | 4.1 | 6.2 | 3.2 |
| 77 | 31.6 | 36.7 | 41.1 | 44.6 | 33.3 | 2.1 | 4.2 | 4.2 | 5.7 | 3.4 |
| 84 | 31.3 | 36.7 | 43.3 | 45.3 | 33.3 | 2.4 | 4.8 | 4.1 | 4.7 | 3.5 |
| 91 | 31.4 | 36.6 | 44.7 | 45.3 | 33.5 | 2.8 | 4.6 | 3.9 | 4.5 | 3.9 |
| 98 | 31.3 | 35.6 | 45.2 | 45.0 | 33.0 | 2.8 | 4.8 | 3.5 | 4.4 | 3.4 |
| 105 | 30.9 | 35.7 | 46.2 | 45.7 | 33.3 | 2.4 | 4.7 | 4.0 | 4.2 | 3.2 |
| 112 | 30.7 | 35.5 | 47.5 | 46.8 | 33.5 | 1.8 | 5.0 | 3.7 | 3.9 | 3.1 |
| 119 | 31.4 | 35.7 | 47.4 | 47.0 | 33.6 | 1.9 | 4.3 | 3.8 | 3.7 | 2.3 |
| 126 | 30.8 | 34.9 | 47.7 | 47.1 | 33.1 | 2.0 | 4.1 | 3.1 | 3.6 | 2.9 |
| 133 | 31.2 | 35.0 | 48.2 | 47.3 | 33.3 | 2.0 | 4.0 | 2.9 | 3.8 | 3.1 |
| 140 | 31.1 | 35.4 | 48.8 | 47.8 | 33.6 | 2.0 | 4.0 | 2.6 | 3.8 | 2.8 |
| 147 | 31.1 | 35.5 | 43.6 | 42.4 | 33.4 | 2.0 | 3.9 | 3.7 | 4.4 | 2.9 |
| 154 | 31.2 | 35.4 | 41.3 | 39.4 | 33.9 | 2.0 | 4.5 | 3.8 | 5.3 | 2.8 |
| 161 | 31.3 | 35.4 | 39.1 | 37.8 | 33.7 | 1.9 | 4.3 | 4.5 | 5.1 | 3.1 |
| 168 | 31.6 | 34.6 | 38.6 | 37.2 | 33.6 | 2.3 | 4.2 | 4.8 | 4.2 | 3.2 |
| 175 | 31.8 | 35.3 | 38.4 | 36.6 | 33.7 | 2.6 | 3.3 | 4.5 | 4.6 | 3.0 |
| 182 | 32.6 | 36.7 | 39.0 | 37.2 | 34.8 | 2.1 | 3.9 | 5.3 | 4.8 | 2.9 |
| 189 | 32.5 | 36.2 | 39.0 | 37.0 | 34.3 | 2.3 | 4.0 | 5.3 | 4.6 | 2.9 |
| 196 | 32.6 | 36.0 | 39.7 | 37.3 | 34.5 | 2.3 | 4.3 | 5.8 | 5.0 | 3.1 |
| 203 | 33.0 | 36.6 | 39.8 | 37.5 | 35.3 | 2.2 | 5.1 | 6.5 | 5.1 | 3.3 |
| 210 | 33.5 | 36.9 | 40.0 | 37.7 | 35.3 | 2.4 | 4.9 | 7.0 | 4.9 | 3.8 |
| 217 | 34.0 | 37.4 | 41.3 | 38.7 | 36.1 | 2.6 | 5.1 | 6.9 | 5.4 | 3.8 |
| 224 | 35.2 | 38.8 | 42.7 | 39.6 | 36.9 | 3.0 | 5.5 | 7.3 | 5.1 | 4.0 |
