# Supplementary material for: Predicting Changes of Body Weight, Body Fat, Energy Expenditure and Metabolic Fuel Selection in C57BL/6 Mice
Source: PLoS One. 2011 Jan 5;6(1):e15961. doi: 10.1371/journal.pone.0015961 (PMC3016341; doi:10.1371/journal.pone.0015961)
Supplement: Table S2 — Body fat measurements and 95% confidence intervals. (DOC) [file pone.0015961.s002.doc]

| day | FM, g |  |  |  |  | 95% CI of FM, g | | |  |  |
| --- | --- | --- | --- | --- | --- | --- | --- | --- | --- | --- |
|  | Control | 7HF-C | HF-C-HF-C | 20HF-C | 4HF-C | Control | 7HF-C | HF-C-HF-C | 20HF-C | 4HF-C |
| 0 | 2.2 | 1.7 | 1.7 | 2.3 | 2.1 | 0.4 | 0.7 | 0.7 | 0.5 | 0.8 |
| 7 | 2.6 | 6.0 | 6.7 | 6.4 | 6.2 | 0.6 | 2.0 | 1.4 | 1.6 | 2.1 |
| 14 | 3.1 | 8.5 | 9.3 | 8.7 | 8.5 | 0.6 | 3.4 | 1.5 | 2.4 | 2.7 |
| 21 | 3.4 | 10.5 | 11.6 | 10.8 | 10.4 | 0.8 | 3.6 | 1.4 | 3.1 | 3.1 |
| 28 | 4.0 | 12.7 | 13.6 | 12.6 | 12.0 | 0.8 | 4.0 | 1.6 | 3.7 | 3.5 |
| 35 | 4.5 | 13.9 | 14.5 | 14.0 | 7.7 | 1.1 | 4.1 | 2.0 | 3.5 | 3.3 |
| 42 | 4.8 | 15.1 | 15.8 | 15.0 | 6.3 | 1.2 | 4.4 | 2.2 | 3.5 | 2.5 |
| 49 | 5.3 | 16.4 | 17.0 | 15.6 | 6.1 | 1.2 | 4.2 | 2.2 | 3.7 | 2.5 |
| 56 | 5.3 | 12.3 | 12.9 | 16.4 | 5.6 | 0.9 | 4.2 | 2.2 | 4.1 | 2.5 |
| 63 | 5.3 | 10.2 | 10.6 | 16.5 | 5.6 | 1.0 | 3.9 | 2.3 | 4.3 | 2.9 |
| 70 | 5.6 | 9.2 | 9.9 | 16.7 | 5.6 | 1.1 | 4.0 | 2.2 | 4.1 | 3.0 |
| 77 | 5.6 | 8.6 | 14.0 | 17.0 | 5.7 | 0.9 | 4.2 | 2.1 | 3.7 | 3.2 |
| 84 | 5.6 | 8.5 | 16.1 | 17.5 | 5.7 | 1.5 | 4.5 | 2.0 | 2.8 | 3.3 |
| 91 | 5.6 | 8.4 | 17.3 | 17.4 | 5.8 | 1.4 | 4.4 | 1.8 | 2.5 | 3.5 |
| 98 | 5.3 | 7.7 | 17.7 | 17.1 | 5.3 | 1.4 | 4.5 | 1.4 | 2.3 | 3.2 |
| 105 | 5.1 | 7.9 | 18.8 | 17.7 | 5.3 | 1.1 | 4.5 | 1.4 | 2.4 | 3.0 |
| 112 | 4.9 | 7.7 | 19.3 | 18.1 | 5.4 | 0.8 | 4.9 | 1.5 | 2.0 | 3.1 |
| 119 | 5.3 | 7.6 | 19.2 | 18.4 | 5.7 | 0.9 | 3.9 | 1.4 | 1.6 | 2.7 |
| 126 | 4.9 | 7.4 | 19.5 | 18.6 | 5.4 | 0.9 | 4.1 | 1.0 | 1.6 | 2.8 |
| 133 | 4.9 | 7.3 | 19.5 | 18.3 | 5.4 | 1.1 | 3.9 | 1.1 | 1.8 | 2.9 |
| 140 | 5.0 | 7.4 | 19.7 | 18.5 | 5.7 | 1.2 | 3.7 | 0.8 | 1.7 | 2.8 |
| 147 | 4.9 | 7.4 | 14.9 | 13.2 | 5.5 | 1.3 | 3.9 | 1.5 | 3.0 | 2.7 |
| 154 | 4.8 | 7.3 | 12.7 | 10.7 | 5.5 | 1.1 | 3.9 | 1.7 | 3.5 | 2.7 |
| 161 | 5.1 | 7.7 | 10.9 | 9.6 | 5.7 | 1.0 | 4.0 | 2.2 | 3.3 | 2.8 |
| 168 | 5.3 | 7.5 | 10.3 | 8.7 | 5.8 | 1.2 | 3.9 | 2.7 | 2.9 | 2.9 |
| 175 | 5.4 | 7.3 | 10.4 | 8.5 | 5.8 | 1.5 | 3.5 | 2.7 | 3.1 | 2.9 |
| 182 | 5.9 | 8.2 | 10.6 | 8.9 | 6.3 | 1.4 | 3.7 | 3.4 | 3.3 | 2.9 |
| 189 | 6.0 | 7.9 | 10.6 | 8.4 | 6.0 | 1.3 | 3.9 | 3.5 | 3.5 | 2.6 |
| 196 | 5.9 | 7.8 | 10.9 | 8.6 | 6.3 | 1.4 | 4.1 | 3.6 | 3.5 | 2.9 |
| 203 | 6.5 | 8.4 | 11.3 | 9.0 | 6.9 | 1.3 | 4.5 | 4.2 | 3.7 | 3.2 |
| 210 | 6.8 | 8.6 | 11.5 | 9.1 | 7.1 | 1.4 | 4.5 | 4.3 | 3.6 | 3.3 |
| 217 | 7.3 | 9.3 | 12.2 | 10.1 | 7.8 | 1.6 | 4.9 | 4.6 | 3.9 | 3.5 |
| 224 | 8.2 | 10.3 | 13.4 | 10.9 | 8.4 | 1.5 | 5.0 | 4.8 | 3.9 | 3.9 |
