# Supplementary material for: Predicting Changes of Body Weight, Body Fat, Energy Expenditure and Metabolic Fuel Selection in C57BL/6 Mice
Source: PLoS One. 2011 Jan 5;6(1):e15961. doi: 10.1371/journal.pone.0015961 (PMC3016341; doi:10.1371/journal.pone.0015961)
Supplement: Table S3 — Energy intake measurements. (DOC) [file pone.0015961.s003.doc]

| day | Energy Intake, kcal/d | | |  |  |
| --- | --- | --- | --- | --- | --- |
|  | Control | 7HF-C | HF-C-HF-C | 20HF-C | 4HF-C |
| 0 | 12.8 | 17.4 | 17.2 | 16.6 | 17.0 |
| 2 | 12.1 | 20.7 | 20.9 | 20.3 | 20.1 |
| 3 | 10.9 | 16.9 | 16.3 | 17.6 | 15.4 |
| 4 | 14.3 | 19.3 | 16.7 | 17.3 | 15.6 |
| 5 | 12.9 | 16.7 | 16.8 | 16.3 | 16.0 |
| 6 | 11.5 | 15.5 | 16.3 | 15.3 | 14.9 |
| 7 | 12.7 | 14.0 | 14.1 | 14.1 | 14.4 |
| 8 | 11.7 | 14.2 | 13.7 | 14.2 | 13.6 |
| 9 | 12.7 | 14.5 | 14.3 | 13.9 | 14.5 |
| 11 | 10.9 | 15.0 | 14.6 | 13.1 | 14.8 |
| 12 | 11.3 | 13.9 | 14.3 | 13.6 | 13.1 |
| 13 | 12.0 | 14.5 | 14.9 | 14.1 | 14.4 |
| 14 | 13.4 | 14.2 | 13.4 | 14.2 | 14.3 |
| 15 | 11.4 | 13.5 | 13.4 | 13.4 | 13.7 |
| 16 | 11.3 | 13.8 | 13.8 | 14.0 | 13.7 |
| 18 | 11.5 | 14.9 | 14.2 | 14.3 | 13.5 |
| 19 | 12.6 | 14.0 | 14.1 | 13.9 | 13.0 |
| 20 | 10.9 | 14.6 | 14.5 | 14.5 | 14.2 |
| 21 | 13.1 | 13.8 | 12.9 | 13.7 | 13.4 |
| 22 | 12.1 | 13.0 | 12.1 | 12.5 | 12.3 |
| 23 | 11.2 | 14.0 | 14.1 | 13.1 | 14.3 |
| 24 | 11.9 | 13.1 | 14.0 | 13.9 | 12.5 |
| 28 | 12.9 | 15.3 | 13.1 | 13.2 | 14.1 |
| 29 | 10.5 | 12.5 | 14.7 | 12.3 | 5.1 |
| 30 | 13.6 | 13.5 | 12.5 | 12.3 | 5.6 |
| 31 | 11.3 | 15.1 | 15.0 | 13.7 | 7.8 |
| 35 | 13.0 | 14.1 | 13.3 | 12.3 | 11.3 |
| 36 | 13.1 | 13.4 | 14.9 | 13.6 | 9.6 |
| 37 | 10.7 | 13.7 | 13.0 | 13.4 | 10.4 |
| 38 | 11.4 | 15.3 | 15.0 | 12.5 | 11.6 |
| 39 | 11.5 | 13.4 | 13.0 | 12.2 | 10.9 |
| 40 | 9.9 | 15.8 | 14.5 | 13.6 | 11.9 |
| 41 | 12.6 | 13.4 | 12.7 | 12.9 | 10.2 |
| 42 | 11.3 | 12.9 | 12.7 | 11.6 | 11.0 |
| 43 | 12.8 | 15.4 | 14.3 | 13.1 | 12.5 |
| 44 | 12.9 | 13.6 | 15.0 | 12.1 | 12.2 |
| 45 | 10.1 | 13.1 | 13.1 | 12.3 | 11.5 |
| 46 | 10.5 | 13.7 | 14.5 | 13.1 | 12.6 |
| 47 | 13.8 | 15.6 | 12.3 | 12.7 | 11.0 |
| 48 | 12.7 | 12.9 | 14.7 | 12.2 | 11.8 |
| 49 | 12.8 | 15.7 | 15.2 | 13.1 | 15.8 |
| 50 | 14.1 | 6.1 | 5.6 | 12.5 | 13.2 |
| 51 | 12.6 | 6.3 | 5.6 | 12.9 | 12.6 |
| 52 | 11.9 | 8.1 | 7.9 | 13.8 | 13.1 |
| 53 | 11.0 | 9.5 | 8.2 | 13.2 | 14.1 |
| 54 | 12.2 | 8.7 | 7.0 | 13.7 | 13.0 |
| 55 | 13.0 | 8.0 | 7.6 | 13.0 | 12.2 |
| 56 | 14.0 | 9.5 | 9.6 | 13.6 | 14.0 |
| 57 | 11.4 | 10.5 | 9.7 | 12.9 | 13.3 |
| 58 | 12.1 | 10.5 | 9.9 | 12.3 | 12.9 |
| 59 | 12.1 | 9.6 | 9.3 | 12.8 | 11.8 |
| 61 | 13.8 | 10.6 | 9.3 | 14.2 | 12.6 |
| 62 | 11.2 | 10.9 | 10.0 | 13.1 | 12.5 |
| 63 | 13.4 | 11.5 | 9.4 | 14.0 | 13.4 |
| 64 | 12.3 | 9.8 | 10.4 | 12.6 | 11.9 |
| 65 | 13.8 | 11.2 | 10.0 | 13.8 | 12.0 |
| 66 | 12.3 | 12.4 | 11.1 | 13.5 | 14.6 |
| 67 | 11.0 | 13.8 | 13.6 | 12.9 | 14.9 |
| 68 | 10.5 | 12.4 | 11.1 | 13.2 | 13.1 |
| 69 | 11.5 | 14.2 | 13.1 | 12.9 | 12.4 |
| 70 | 13.6 | 13.4 | 21.8 | 14.2 | 13.4 |
| 71 | 11.4 | 13.2 | 16.0 | 12.4 | 12.7 |
| 72 | 13.4 | 14.8 | 19.8 | 13.7 | 14.6 |
| 73 | 13.2 | 11.6 | 16.1 | 12.5 | 12.4 |
| 74 | 13.3 | 13.4 | 17.7 | 13.4 | 15.0 |
| 75 | 13.4 | 13.7 | 17.4 | 13.1 | 13.8 |
| 76 | 14.6 | 10.8 | 15.4 | 13.7 | 12.8 |
| 77 | 13.7 | 11.5 | 16.3 | 13.8 | 14.6 |
| 78 | 13.6 | 13.1 | 16.3 | 14.6 | 13.8 |
| 79 | 11.7 | 15.3 | 16.3 | 13.8 | 15.0 |
| 80 | 13.4 | 14.1 | 14.3 | 13.4 | 13.5 |
| 83 | 13.3 | 12.4 | 15.1 | 12.0 | 14.5 |
| 84 | 12.5 | 12.7 | 14.8 | 14.2 | 13.5 |
| 85 | 12.4 | 13.4 | 14.1 | 13.4 | 15.4 |
| 86 | 13.4 | 12.3 | 12.6 | 11.9 | 14.5 |
| 90 | 9.9 | 15.5 | 16.6 | 14.6 | 13.8 |
| 91 | 10.8 | 12.8 | 15.5 | 13.0 | 15.1 |
| 92 | 13.4 | 13.8 | 12.7 | 14.5 | 14.2 |
| 93 | 12.4 | 12.1 | 12.8 | 12.9 | 15.4 |
| 96 | 11.8 | 15.4 | 14.4 | 14.5 | 12.8 |
| 97 | 11.8 | 14.3 | 13.8 | 13.1 | 14.4 |
| 98 | 13.1 | 11.7 | 15.0 | 14.2 | 15.0 |
| 99 | 13.0 | 16.2 | 13.3 | 13.3 | 13.0 |
| 104 | 11.6 | 12.8 | 15.1 | 13.9 | 15.0 |
| 105 | 12.6 | 12.3 | 14.1 | 14.5 | 13.3 |
| 106 | 12.4 | 16.3 | 13.4 | 13.9 | 14.5 |
| 107 | 11.5 | 12.7 | 13.6 | 14.3 | 12.8 |
| 110 | 14.1 | 16.7 | 15.6 | 13.9 | 12.7 |
| 111 | 13.3 | 12.8 | 12.7 | 12.5 | 13.5 |
| 112 | 11.8 | 14.9 | 13.8 | 14.8 | 15.0 |
| 113 | 14.1 | 17.3 | 14.2 | 13.9 | 12.8 |
| 114 | 13.2 | 12.7 | 12.4 | 13.4 | 14.2 |
| 117 | 12.0 | 14.2 | 14.0 | 14.4 | 14.3 |
| 118 | 12.2 | 17.3 | 14.4 | 13.7 | 12.9 |
| 119 | 13.5 | 12.7 | 15.5 | 14.7 | 14.1 |
| 120 | 14.0 | 16.9 | 14.6 | 14.4 | 14.9 |
| 121 | 12.8 | 15.0 | 13.8 | 13.8 | 13.4 |
| 124 | 11.8 | 14.1 | 14.8 | 14.2 | 14.0 |
| 125 | 13.7 | 12.6 | 13.0 | 13.5 | 12.9 |
| 126 | 14.3 | 16.7 | 15.3 | 14.1 | 15.4 |
| 127 | 13.4 | 13.8 | 13.5 | 13.2 | 13.8 |
| 131 | 13.5 | 16.4 | 15.1 | 14.2 | 14.1 |
| 132 | 12.5 | 12.8 | 13.8 | 13.2 | 14.7 |
| 133 | 13.8 | 15.9 | 14.7 | 13.9 | 14.6 |
| 134 | 14.4 | 13.3 | 14.6 | 14.2 | 15.2 |
| 135 | 14.5 | 13.7 | 14.2 | 14.0 | 13.7 |
| 138 | 12.8 | 15.5 | 15.7 | 14.5 | 13.1 |
| 139 | 12.3 | 15.9 | 9.9 | 9.9 | 13.6 |
| 140 | 13.0 | 13.7 | 8.7 | 11.3 | 13.0 |
| 141 | 14.8 | 14.8 | 10.5 | 8.8 | 13.7 |
| 142 | 13.2 | 15.0 | 9.5 | 10.3 | 14.9 |
| 145 | 14.5 | 16.1 | 11.3 | 9.5 | 15.3 |
| 146 | 12.0 | 13.4 | 10.2 | 9.8 | 12.5 |
| 147 | 12.8 | 16.7 | 11.1 | 11.8 | 15.3 |
| 152 | 14.8 | 15.4 | 10.8 | 13.1 | 12.8 |
| 153 | 13.9 | 14.8 | 11.5 | 13.2 | 15.0 |
| 154 | 12.6 | 16.3 | 10.8 | 13.8 | 14.2 |
| 159 | 12.8 | 14.1 | 12.2 | 12.3 | 12.9 |
| 160 | 15.8 | 15.9 | 11.4 | 11.3 | 14.5 |
| 161 | 13.8 | 15.2 | 12.6 | 11.7 | 13.9 |
| 163 | 15.1 | 16.4 | 11.8 | 15.1 | 15.1 |
| 167 | 13.0 | 13.4 | 13.9 | 13.7 | 13.0 |
| 168 | 15.1 | 15.0 | 15.4 | 14.2 | 15.5 |
| 169 | 12.6 | 12.8 | 12.4 | 12.3 | 12.6 |
| 170 | 14.7 | 15.8 | 16.3 | 15.2 | 15.2 |
| 173 | 13.1 | 12.5 | 13.5 | 13.3 | 14.0 |
| 175 | 14.1 | 16.5 | 17.1 | 15.3 | 14.4 |
| 176 | 15.8 | 14.3 | 16.2 | 13.8 | 13.5 |
| 177 | 12.8 | 15.9 | 14.6 | 15.1 | 15.0 |
| 182 | 14.0 | 16.2 | 16.1 | 14.8 | 14.2 |
| 183 | 14.0 | 13.1 | 13.8 | 11.6 | 14.5 |
| 184 | 14.0 | 16.8 | 15.0 | 13.7 | 14.9 |
| 187 | 14.1 | 12.9 | 15.4 | 14.0 | 13.7 |
| 188 | 15.0 | 14.5 | 14.5 | 16.3 | 14.0 |
| 189 | 12.7 | 16.1 | 16.3 | 16.9 | 15.2 |
| 190 | 15.5 | 14.2 | 13.2 | 15.1 | 14.5 |
| 191 | 13.5 | 13.3 | 14.3 | 14.2 | 13.7 |
| 194 | 16.0 | 16.0 | 17.9 | 16.8 | 15.6 |
| 196 | 15.3 | 13.9 | 13.8 | 14.4 | 14.4 |
| 197 | 15.3 | 16.3 | 15.4 | 15.1 | 15.5 |
| 198 | 15.4 | 15.0 | 15.3 | 16.1 | 15.7 |
| 201 | 14.9 | 16.5 | 16.8 | 16.5 | 15.3 |
| 202 | 13.5 | 15.8 | 16.0 | 15.5 | 16.1 |
| 203 | 12.5 | 14.1 | 15.2 | 14.6 | 14.9 |
| 204 | 16.7 | 12.7 | 13.5 | 13.9 | 15.2 |
| 205 | 15.0 | 13.4 | 14.8 | 13.5 | 14.5 |
| 208 | 11.9 | 18.0 | 17.0 | 16.9 | 16.2 |
| 209 | 17.2 | 14.8 | 15.1 | 16.0 | 14.7 |
| 210 | 14.6 | 12.8 | 13.4 | 13.2 | 13.8 |
| 211 | 15.1 | 16.7 | 16.8 | 17.3 | 15.8 |
| 212 | 16.2 | 16.5 | 16.5 | 17.2 | 15.2 |
| 215 | 13.1 | 13.6 | 17.7 | 14.9 | 14.3 |
| 216 | 14.0 | 15.6 | 18.7 | 17.6 | 15.5 |
| 217 | 14.8 | 13.5 | 13.9 | 14.8 | 16.0 |
| 218 | 12.9 | 13.6 | 14.8 | 14.1 | 13.3 |
| 222 | 14.3 | 15.6 | 16.8 | 14.5 | 15.5 |
| 223 | 16.3 | 13.1 | 14.2 | 13.6 | 13.7 |
| 224 | 13.4 | 14.0 | 14.1 | 14.5 | 14.5 |
| 225 | 15.0 | 16.7 | 17.5 | 16.9 | 16.1 |
| 226 | 14.0 | 14.3 | 14.8 | 15.3 | 13.7 |
| 229 | 12.3 | 15.6 | 15.1 | 14.9 | 13.7 |
| 230 | 14.0 | 14.3 | 16.1 | 14.9 | 12.7 |
| 231 | 13.9 | 12.2 | 13.4 | 13.4 | 12.0 |
